# Optimization of Deep Learning Parameters for Magneto-Impedance Sensor in Metal Detection and Classification

**DOI:** 10.3390/s23229259

**Published:** 2023-11-18

**Authors:** Hoijun Kim, Hobyung Chae, Soonchul Kwon, Seunghyun Lee

**Affiliations:** 1 Department of Plasma Bio Display, Kwangwoon University, 20 Kwangwoon-ro, Seoul 01897, Republic of Korea; 2 Industry-Academic Cooperation Foundation, Kwangwoon University, 20 Kwangwoon-ro, Seoul 01897, Republic of Korea; 3 Department of Smart Convergence, Kwangwoon University, 20 Kwangwoon-ro, Seoul 01897, Republic of Korea; 4 Ingenium College of Liberal Arts, Kwangwoon University, 20 Kwangwoon-ro, Seoul 01897, Republic of Korea

**Keywords:** CNN, deep learning, metal detection, MI sensor, RNN

## Abstract

Deep learning technology is generally applied to analyze periodic data, such as the data of electromyography (EMG) and acoustic signals. Conversely, its accuracy is compromised when applied to the anomalous and irregular nature of the data obtained using a magneto-impedance (MI) sensor. Thus, we propose and analyze a deep learning model based on recurrent neural networks (RNNs) optimized for the MI sensor, such that it can detect and classify data that are relatively irregular and diverse compared to the EMG and acoustic signals. Our proposed method combines the long short-term memory (LSTM) and gated recurrent unit (GRU) models to detect and classify metal objects from signals acquired by an MI sensor. First, we configured various layers used in RNN with a basic model structure and tested the performance of each layer type. In addition, we succeeded in increasing the accuracy by processing the sequence length of the input data and performing additional work in the prediction process. An MI sensor acquires data in a non-contact mode; therefore, the proposed deep learning approach can be applied to drone control, electronic maps, geomagnetic measurement, autonomous driving, and foreign object detection.

## 1. Introduction

The burgeoning realm of deep learning has revolutionized various sectors, particularly the image and signal processing fields. Its prowess in object recognition, segmentation, detection, pose estimation, and even face and voice recognition has been well documented and widely acclaimed [1,2,3,4,5,6,7,8,9,10,11,12,13,14,15,16,17,18,19,20,21,22,23,24]. The primary strength of deep learning, especially in signal processing, lies in its adeptness at handling time series data, often spanning single or multiple channels [25,26,27,28,29]. This capability enables it to discern patterns, establish correlations between sequential data points, and significantly enhance processing quality.

Yet, while the accomplishments of deep learning in conventional domains are commendable, its application in more niche sectors, such as the post-processing of magneto-impedance (MI) sensors, remains relatively uncharted [30]. MI sensors, a recent technological innovation, operate based on the magnetic impedance phenomenon. Typically, a ferromagnetic object, often an amorphous wire, carries a high-frequency or pulsed current. When subjected to an external magnetic field, this object undergoes the skin effect [31,32,33,34]. To optimize sensitivity, these sensors often employ a pulsed magnetic field ranging between 0.5 and 1 GHz. Given their sensitivity to magnetic fields, MI sensors have found applications in diverse areas, such as drone control, electronic mapping, geomagnetic measurements, autonomous driving, and, crucially, foreign object detection at security checkpoints.

However, a glaring challenge emerges when processing MI sensor data. Contrary to the periodic and regular signals, such as those from EMG or acoustic sources, MI sensor outputs are characterized by their anomalies and time-variances. This irregularity renders traditional deep learning models, which excel with more structured data, less effective.

Recognizing this gap, our paper embarks on a journey to harness the strengths of deep learning, particularly focusing on recurrent neural networks (RNNs), to tailor a solution optimized for MI sensor data. We aim to pioneer a model capable of navigating the intricacies of MI sensor outputs, offering superior performance in detection and classification tasks, and standing tall when juxtaposed with traditional methods.

## 2. The RNNs

An RNN is a deep learning model that is largely composed of recurrent neurons, memory cells, and input and output sequences and is suitable for continuous time series data such as natural language (NL), speech signals, and stocks [35,36,37,38,39,40]. It processes inputs and outputs in sequence units; wherein, the connection between the units exhibits a cyclic structure. In general, the RNN exhibits the input and output for each time slot by expanding the recurrent neurons for each time slot due to an output being received back as an input in a cyclic method contrary to the feedforward method, which allows a unidirectional processing. For each time slot, every neuron receives an input and an output from the previous time slot. Thus, the output of the recurrent neuron is a function of all inputs in the previous time slot and can be viewed as a memory. A memory cell is a component of a neural network that preserves some state across time slots. The input and output sequences are classified based on the purpose and method of using an RNN. First is the vector-to-sequence method, which is an image captioning method. Second, the sentiment classification method converts a sequence into a vector or a sequence into a sequence. Third is the delayed sequence-to-sequence method which is used for machine translation. In this study, we apply the sentiment classification method to detect and classify metal objects from signals acquired by an MI sensor [41,42].

Such an RNN model faces problems with backpropagation through time (BPTT) and long-term dependency. Because an RNN is backpropagated from beginning to end for every time slot in the learning process, for large time slots, the RNN will be very deep, and the vanishing and exploding gradient problems are highly likely to occur. Additionally, the large computational time increases the learning time. To address this issue, the truncated BPTT method is applied which divides time slots into predetermined sections to approximately evaluate the backpropagation process and ensure effective learning. However, in the RNN model, previous information received can be affected by current information. Thus, all previous time slots are affected, but as the time slot becomes longer, the front side remains unaffected, which is called a long-term dependency problem. Thus, the long short-term memory (LSTM) and gated recurrent unit (GRU) models, which are improved RNN models, have been developed.

### 2.1. LSTM Cell

**Figure 1:**
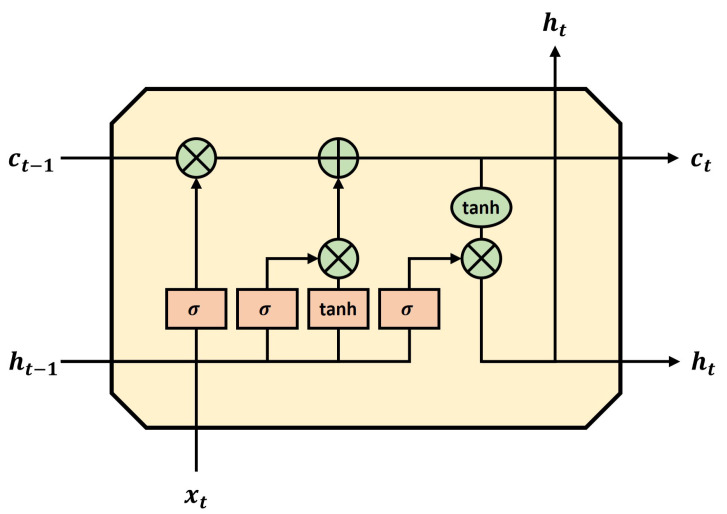
Representation of an LSTM cell. Here, *h* and *c* denote the vectors of the hidden layer, *x* represents the input, *t* stands for the current time step, and t − 1 indicates the previous time step.

An LSTM cell not only solves the long-term dependency problem of RNNs, but its learning also converges swiftly [43,44,45]. The LSTM basically has an RNN structure, and the network learns the part to remember, the part to delete, and the part to read in a long-term state. The long-term dependency problem can be solved because both the long-term and short-term states are preserved and learned. Therefore, long-term state information does not accumulate. In addition, the output range is adjusted from 0 to 1 using sigmoid as the activation function in the three layers inside the LSTM. When the output is 0, the gate is closed, and when the output is 1, the gate is opened to control the output direction. Accordingly, the backpropagation process is controlled, and the BPTT problem is solved. In Figure 1, we delve into the intricacies of the LSTM cell, elucidating its structure and the manner in which data flows through its various components.

### 2.2. GRU Cell

**Figure 2:**
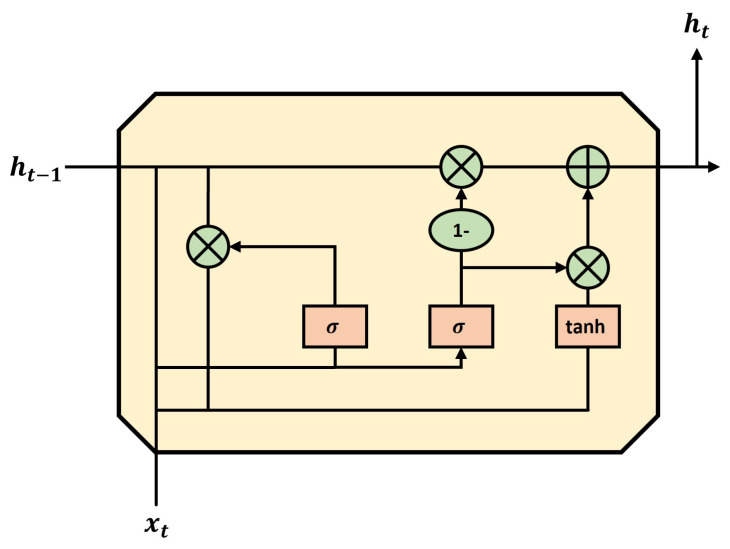
Representation of a GRU cell. In this diagram, *h* denotes the vector of the hidden layer, *x* represents the input, *t* stands for the current time step, and t − 1 indicates the previous time step.

A GRU cell is a simplified model of the LSTM cell. In the LSTM cell, two state vectors are combined into a single vector [46,47,48]. This controls the part to be deleted as well as the part to receive input with one inner layer. Similar to the LSTMs, the output is determined using the sigmoid activation function. When the output is 0, the delete part is closed and the input part is open. When the output is one, the delete gate is opened and the input gate is closed, thereby determining the direction of delivery. Unlike the LSTM, the GRU cell does not have an output part; therefore, the entire state vector is the output for each time slot. A new layer is added to control the outputs of the previous state. The GRU is a simplified and faster version of the LSTM. In this paper, the LSTM and GRU are combined into an advanced model; the accuracy and speed tests were conducted for each layer number and model type. An illustrative depiction of the LSTM cell highlights its intricate mechanisms and flow of information. Moving on to Figure 2, we present the GRU cell, outlining its distinctive architecture and highlighting the dynamics of its functional elements.

## 3. Optimizing Deep Learning Models for Metal Object Detection

### 3.1. Data Acquisition and Dataset Configuration

**Figure 3:**
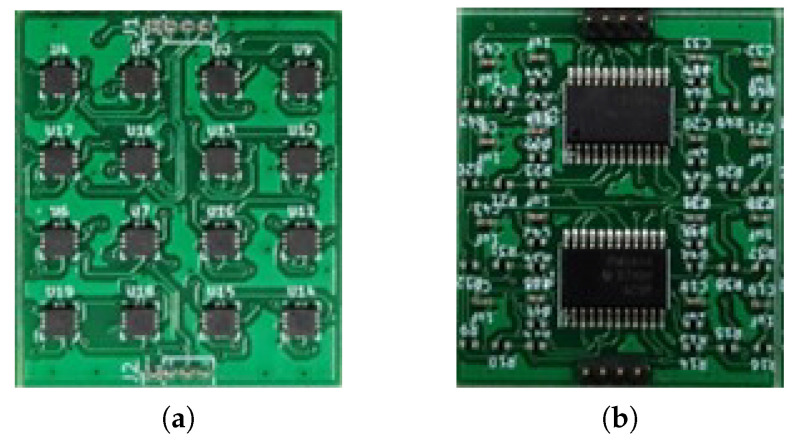
The 4 × 4 array of AMI306 sensors. (**a**) The 16 AMI306 sensors. (**b**) Two MPUs (ARM Cortex-M4).

In this paper, the data were acquired using AICHI”s AMI306 sensor (Aichi Steel Corporation, (1, Wanowari, Arao-machi, Tokai-shi, Aichi-ken 476-8666, Japan)) [32]. Of the x, y, and z axes, only the z-axis was used to determine the data based on the z-axis distance between the sensor and the metal object in various ways. We measured variously by changing the measuring distance and speed. To reduce the error in sensitivity, 16 sensors were used in a 4 × 4 arrangement, as shown in Figure 3. Thus, the sensor obtained data with a reduced error by averaging 16 sensor values. The frequency of the sensor was about 200 Hz. The Ethernet socket communication method was used to transfer the data between the sensor and the computer.

Five types of models of the metal objects, manufactured by flattening a square iron plate, were used in this study. To classify the metal objects by size through a deep learning algorithm, the steel plates were configured in different sizes. Each model was manufactured with the same thickness. Table 1 lists the type, size, and weight of each model.

**Figure 4:**
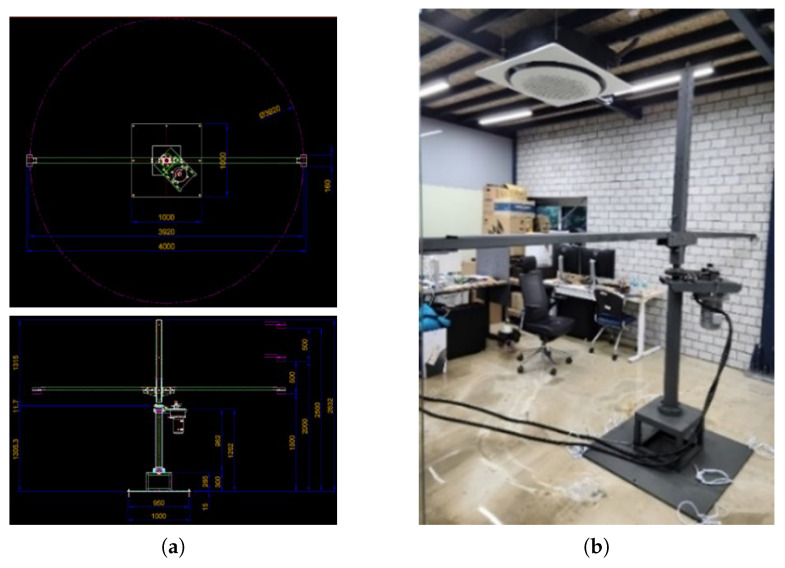
Test fixture for dataset construction. (**a**) Blueprint of test fixture. (**b**) Real test fixture.

A test fixture was constructed to obtain consistent data for the metal model using the MI sensor. It comprises a system that moves in a circle (radius = 2 m) and can always attain a constant speed, of a maximum of 20 m/s, by installing a motor. The data were collected after installing a metal object away from the floor by performing the circular motion at a height of 1.5 m. Figure 4 depicts the test fixture configured to acquire data in this paper.

**Figure 5:**
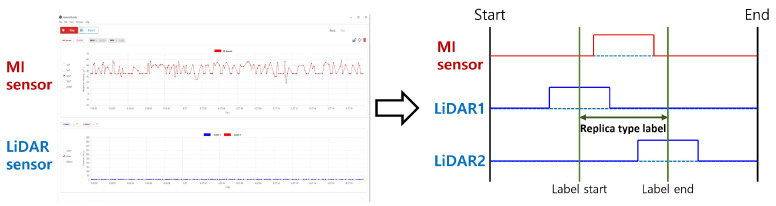
Presentation of the real-time data acquisition and viewer tool for both MI and LiDAR sensors on the left side. On the right, an example demonstrates the labeling of MI sensor values based on LiDAR sensor readings.

A light detection and ranging (LiDAR) sensor (Device name [SJ-PM-TFmini Plus-T-01 A01], Benewake (Beijing) Co., Ltd., Haidian District, Beijing, China) [49,50] was used to accurately label the position of metal objects after data acquisition. Labeling, as represented in Figure 5, was performed based on the information obtained from the LiDAR sensors, which were installed on the front/rear side of the MI sensor. The LiDAR sensor measured the distance between the sensor and the object in the vertical direction. The data on the floor were labeled as 0, and, in the case of classification data, from 1 to 5 for each object, and 1 for the metal object for detection data. The labeling criterion was set as the start and end of the signal of the MI sensor for the metal object with the position of the object detected by the LiDAR sensor as the center.

**Figure 6:**
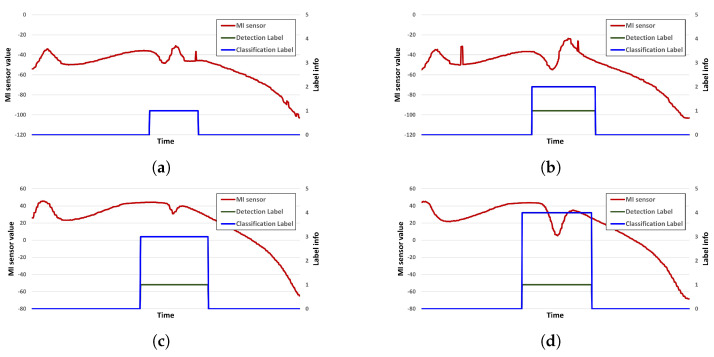
Example labeled data in csv file. (**a**) Plate A, (**b**) plate B, (**c**) plate C, and (**d**) plate D.

For dataset configuration, the measurement speed was set to 1 m/s, 3 m/s, and 5 m/s, and the distance between the sensor and the object was set to 20 cm, 30 cm, and 40 cm, respectively, for each measurement speed. All of the data acquisition frequencies of the MI sensors were the same. The data were acquired 20 times for each distance and speed. There are three distances and speeds, respectively, and a total of nine measurement methods are used. The data were measured 20 times in nine different ways and 180 times for each metal model. The dataset configuration for deep learning and verification was carried out through a total of 900 data acquisition processes. All of the acquired data were in the csv file format. Figure 6 provides a visualization of some of the constructed data.

Figure 6 illustrates the MI sensor data, which are automatically labeled through the LiDAR sensor. The curve-like nature of the data arises because our test rig performs a circular motion. While in operation, the MI sensor retains its default and base signal values, and during this circular motion, influences from the MI sensor cause the recorded values to trace a circular shape. The red line indicates the MI sensor data, the green line is the labeling value acquired by detection, and the blue line is the labeling value obtained through classification. The label value assumes ‘0’ when no metal object is present and ‘1’ when a metal object is present, ensuring accurate detection results. In terms of classification, each type of metal model was assigned a number from ‘1 to 5’, with ‘0’ representing the absence of any model.

### 3.2. Deep Learning Model Configurations

**Figure 7:**
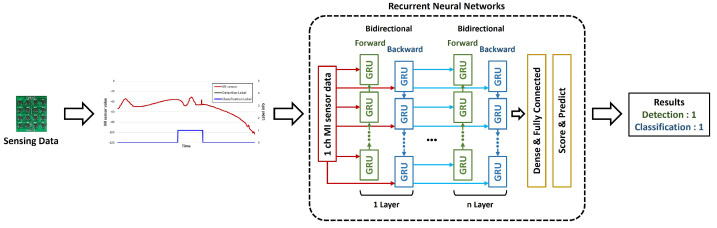
Overall flow for comparison of deep learning algorithms in this paper. Note: The GRU cell can be substituted with an LSTM cell.

The RNN model commonly used in the signal processing field was used as a base. RNN exhibits excellent performance for iterative and continuous data analysis. In this paper, a model was constructed by combining the LSTM and the GRU layers to overcome the shortcomings of conventional RNNs in obtaining accurate data, which is anomalous, over long intervals. Bidirectionality was applied to each layer to enable backward and forward learning of signals. A corresponding layer with one, three, five, seven, and nine number of layers was also applied, and only one type of layer was used in the model each time. This was intended to evaluate a single kind of performance rather than evaluating the complex performance. Figure 7 presents a comparison between the various deep learning algorithms.

**Figure 8:**
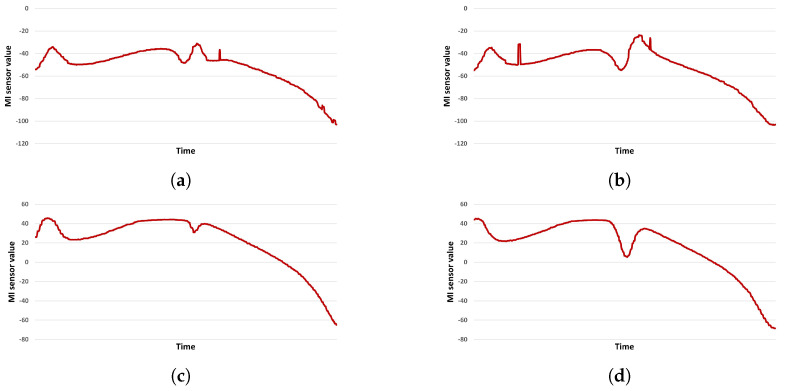
Example input data for an RNN Model. (**a**) Plate A, (**b**) plate B, (**c**) plate C, and (**d**) plate D.

Because raw data were used as the input of the RNN model, the data obtained during the dataset construction process were used without additional pre-processing. Furthermore, when preprocessing the signal processing series, minute changes in the MI sensor data may be determined as noise and removed by filtering. To prevent this, filtering was not performed, and raw MI sensor data were used as input to the RNN model. The signals for the AMI306 sensor (Aichi Steel Corporation, (1, Wanowari, Arao-machi, Tokai-shi, Aichi-ken 476-8666, Japan)) array are included in the csv file. Since the detection and classification processes were performed, there are two types of labeling information detection (presence or absence of metal objects) and classification (labeling of five types of metal models). The data were composed of one sensor signal channel and two labels. Figure 8 gives an example of the input data used to train an RNN model.

Figure 8 provides a visual representation of the typical input data fed into our deep learning model. This data forms the foundation upon which our model predictions are based, ensuring that the model is trained and tested on realistic and representative sequences.

The RNN model is unable to learn data when the time series data are input as one data unit. When the unit of the data is one, the correlation between the current data, the previous data, and the subsequent data cannot be grasped; therefore, the deep learning method of finding the rule only grasps the correlation between the signal for one channel and the labeling data. Accordingly, by adjusting the length of the input data, it is necessary to designate the length of the signal that the RNN model can learn at once. The detection performance varies according to the length of the input data. In this paper, the detection performance based on the length of the input data is analyzed and compared by varying the length of the data.

### 3.3. RNN Model Implementation

An MI sensor performs real-time data acquisition, detection, and classification, as opposed to detecting and classifying accumulated data. In addition, the MI sensor is a low-power consumption sensor, and the learning and verification was carried out based on the CPU considering its portability and use in various environments. The environment in which learning and verification were conducted in this paper are listed in Table 2.

The network comparison experiment was performed to measure and compare the computational time, accuracy, and inference time with respect to the number of layers. The classification accuracy for each metal model and the accuracy of the presence or absence of metal objects were compared and evaluated. The loss function used in RNN training is the L1 loss function (mean absolute error), justified in Equation (1), given as follows:

(1) $$L_{D}=\sum_{i=0}^{n}\left|D_{i}-f_{D}\left(x_{i}\right)\right|$$

(2) $$L_{C}=-\sum_{i=0}^{n}C_{i}\cdot \log\left(f_{C}\left(x_{i}\right)\right)$$

Equation (1) represents the mean absolute error (MAE), which calculates the absolute difference between the predicted results and the ground truth. In this equation, *n* stands for the total number of data samples. $f_{D}$ denotes the detection deep learning network, *x* is the input data, and $D_{i}$ represents the ground truth for detection. The MAE quantifies the discrepancy between the correct detection data and the predictions from the deep learning network.

Equation (2) defines the cross-entropy loss function. This loss function measures the difference between the probability distribution of the predicted results and the actual data. $f_{C}$ should indeed represent the probability distribution outputted by the classification deep learning network, as pointed out by the reviewer. Meanwhile, *x* is the input data, and $C_{i}$ denotes the correct classification labels. This equation computes the discrepancy between the correct classification labels and the predictions from the deep learning network.

Among the training parameters, the optimizer was set to Adam, epoch 300, and batch size 200 to proceed with learning.

### 3.4. Time Slot Analysis

**Figure 9:**
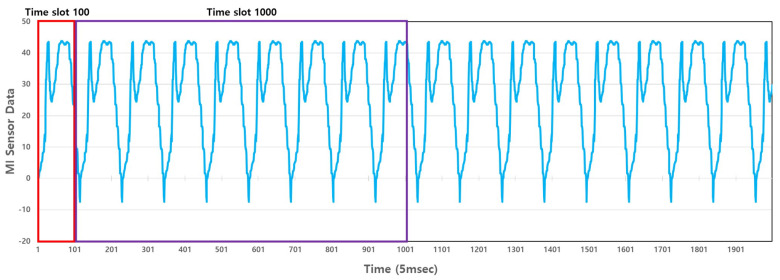
Example of time slot in a time series signal.

We compared the performance of RNN models according to time series units. The RNN model for analyzing time series data learns by grouping data based on their length rather than learning one by one. Accordingly, the continuity and interrelationship of signals are learned. This constant length is called a time slot, and the learning length affects the accuracy of the RNN model. If the time series unit is shorter than the period of the signal, the regularity of the signal may become ambiguous, and the accuracy may be lowered. In this paper, the optimal time series unit was analyzed by varying the length of the time series unit. Figure 9 presents an example of the time series unit.

In Figure 8, we visually represent how varying time series units can affect the representation of signals. As the length of the time series unit changes, different portions of the signal are captured, highlighting the importance of selecting an optimal time slot for RNN learning. If the time slot is too short, significant patterns within the signal might be missed. On the other hand, if it is too long, the model might be overwhelmed with unnecessary details, potentially obscuring the critical patterns.

Furthermore, Figure 8 serves as a visual aid for understanding the practical implications of our discussion on time series units. It provides a tangible representation of how the same signal can be perceived differently based on the chosen time series unit, thereby emphasizing the necessity to optimize this parameter for accurate RNN modeling.

**Figure 10:**
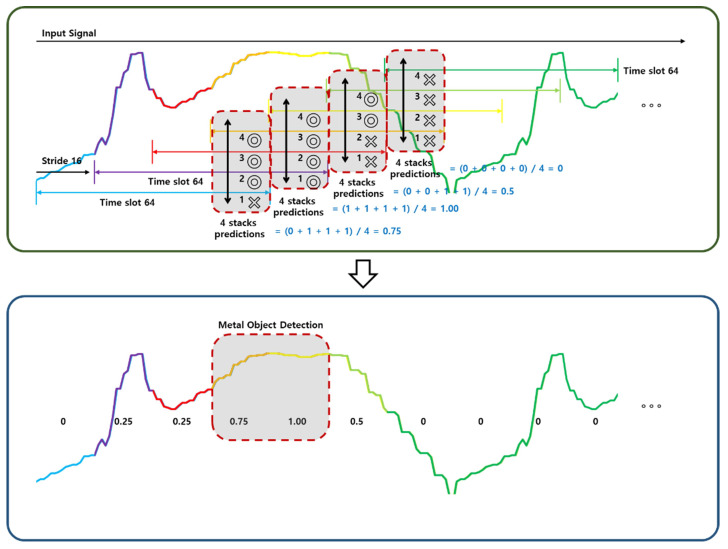
Schematic of nested prediction methods.

Our technique is designed to improve the accuracy of predicting input data by performing multiple overlapping predictions for each region of interest. The key idea behind our approach is to increase the initial point of the time slot by 1/4 of the time slot to overlap the prediction range. Figure 10 illustrates a superposition prediction method, which is our proposed post-processing method.

To be more specific, we divide the input data into regions of interest and perform up to four overlapping predictions for each region. The double circles represent instances where the deep learning model infers the presence of a metallic object, while the ’X’ denotes its absence. For each signal region, detection is performed four times. This is achieved by moving a filter with a length of a quarter of the predicted signal, resulting in four detections for a single area.

For instance, if the time slot size is *T* seconds, we would perform four predictions for a region of interest, with the initial points of the time slots shifted by T/4 seconds each. This way, each prediction overlaps with the previous and next predictions by 3T/4 seconds.

For each prediction, we use a detection and classification algorithm to identify objects in the region of interest. The detection algorithm identifies potential objects by analyzing the image or signal data, while the classification algorithm assigns labels to the objects based on their characteristics. By performing multiple predictions, we increase the chances of detecting and classifying objects accurately.

Once we have obtained the detection and classification outputs for all four predictions, we compare them. Hence, if all four detections indicate the presence of a metal object, it is marked as ’1’. If one detection is absent, it is marked ’0.75’; two absences are marked ’0.5’, three absences are marked ’0.25’, and, if all four detections indicate absence, it is marked ’0’. In other words, we only report the result if it is consistent across multiple predictions.

Our approach has several advantages over the single prediction method. First, it significantly improves the accuracy of detection and classification outputs by performing up to four overlapping predictions for one region of interest. Second, it enables us to detect and classify objects that might have been missed in a single prediction due to noise or other factors. Finally, it provides a level of confidence in the prediction results, as we only report a result if it is consistent across multiple predictions.

However, the trade-off is that the prediction time of the entire data is increased by a factor of four due to the multiple predictions. Therefore, our technique is most suitable for applications where accuracy is paramount and where prediction time is not a critical factor.

## 4. Experimental Results

### 4.1. Evaluation Index and Deep Learning Model Parameters

The dataset was constructed with a total of 900 datasets by measuring the distance of metal objects from the sensor (20 cm, 30 cm, 40 cm) for five types of metal objects. Set the sensor movement speed to 1 m/s, 3 m/s, and 5 m/s for 20 measurements each. The training data and test data were used in an 8:2 ratio. The time slot unit of the input time series data for prediction and learning was set to 64. It was compared and analyzed using the accuracy index of Equation (3) to evaluate the learned model.

(3) $$Accuracy=\frac{Number\;of\;Correct\;Predictions}{Total\;Number\;of\;Predictions}\times 100$$

Accuracy is computed by dividing the number of correct predictions by the total number of predictions.

Table 3 lists the number of parameters and inference time based on the RNN layer type. Compared to the CNN model that processes images, the RNN model requires fewer parameters and exhibits a faster inference time. There are four layers: LSTM, LSTM-Bidirectional, GRU, and GRU-Bidirectional. In addition to this, there are various layers, such as the embedding layer, which we have not used because they are used for natural language processing or are more suitable for other purposes.

Overall, the general LSTM and GRU models have more parameters than the bidirectional model, and the inference time is also relatively slow. The GRU-Bidirectional model has fewer parameters than other models for all of the number of layers, and the inference time was the fastest. The inference time of the models is the lowest when the time slot of the input data is 64, and the nine-layer model of LSTM, which has the slowest inference time, can process in real time up to a sensor data acquisition frequency of up to about 43,000 Hz. The fastest model, the one-layer GRU-Bidirectional, is capable of real-time prediction up to about 92,000 Hz.

### 4.2. Performance Comparison Based on the RNN Layer Type

**Figure 11:**
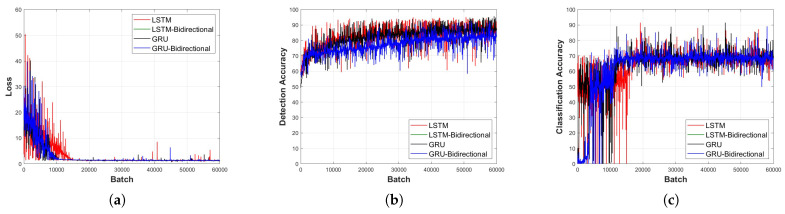
Learning convergence graph for each layer type (depth = 1 layer). (**a**) Loss convergence graph, (**b**) detection accuracy convergence graph, and (**c**) classification accuracy convergence graph.

**Figure 12:**
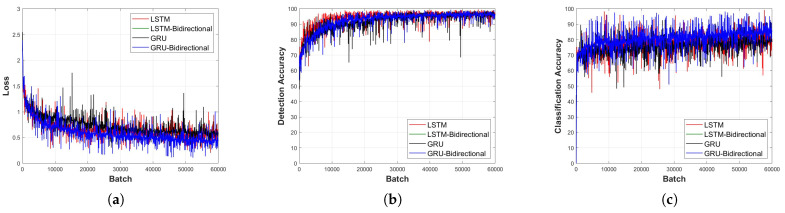
Learning convergence graph for each layer type (depth = 3 layers). (**a**) Loss convergence graph, (**b**) detection accuracy convergence graph, and (**c**) classification accuracy convergence graph.

**Figure 13:**
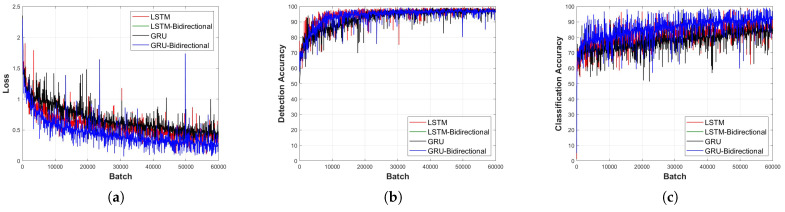
Learning convergence graph for each layer type (depth = 5 layers). (**a**) Loss convergence graph, (**b**) detection accuracy convergence graph, and (**c**) classification accuracy convergence graph.

**Figure 14:**
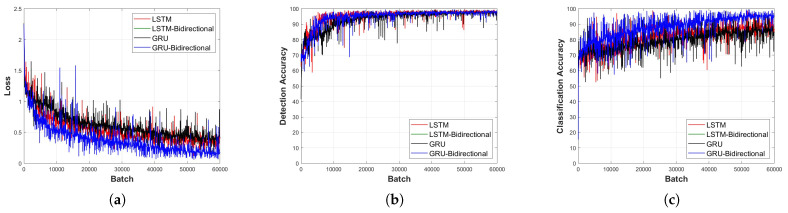
Learning convergence graph for each layer type (depth = 7 layers). (**a**) Loss convergence graph, (**b**) detection accuracy convergence graph, and (**c**) classification accuracy convergence graph.

**Figure 15:**
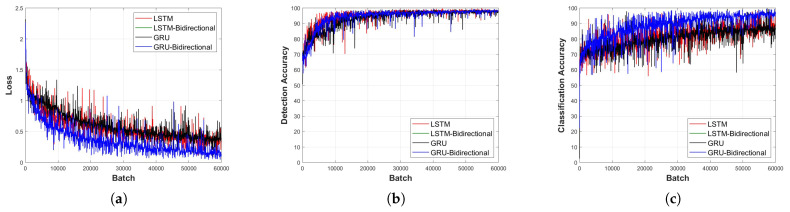
Learning convergence graph for each layer type (depth = 9 layers). (**a**) Loss convergence graph, (**b**) detection accuracy convergence graph, and (**c**) classification accuracy convergence graph.

Figure 11, Figure 12, Figure 13, Figure 14 and Figure 15 depict the loss function and accuracy convergence graphs according to the type and depth of each layer of RNN when the distance between the sensor and the object is 20 cm. All RNN models converge in a similar manner, and the GRU model exhibited the best performance. When the layer was too shallow, data learning was not performed smoothly, and loss and accuracy were unstable at the beginning of learning. Subsequent to converging, the predicted results tended to overfit the training data. When the layer was deep, fast convergence, stability, and accuracy were shown to be high.

In the learning loss and accuracy convergence graph, the LSTM layer, when set to a single layer depth, exhibits optimal performance. This is indicative of the balance between model complexity and its ability to learn the underlying features of the data. Notably, with a shallow model depth, we observed increased fluctuations in the loss value, decreased accuracy, and slower convergence. This behavior suggests that a model with insufficient depth might struggle to capture the intricate features of the signal. Conversely, as the depth increased, the GRU-Bidirectional model outperformed others, demonstrating rapid convergence and superior performance. Such observations underline the significance of model depth and architecture in determining the learning capabilities of RNNs.

It is also worth noting that the detection models generally exhibited faster convergence and higher accuracy compared to classification models. This could be attributed to the inherent challenges associated with multi-class classification tasks, especially when dealing with intricate signal patterns.

Table 4 offers a comprehensive performance comparison across the test set, factoring in the varying layer types and depths of the RNN. It sheds light on two key performance metrics: detection, which determines the presence or absence of a metal object, and classification, which recognizes and categorizes among five distinct metal models. This table serves as a testament to the varying capabilities of different RNN configurations and provides insights into their respective strengths and limitations.

Moreover, it is crucial to emphasize that while certain RNN configurations might excel in one aspect, they might not necessarily be the best fit for other tasks. For instance, while GRU-Bidirectional models might converge faster and demonstrate lower loss values, they might require more computational resources. Such trade-offs should be considered when selecting an appropriate model for specific applications.

The model was hardly trained in the first layer of the four models, most of the signals were predicted as 0, and it was confirmed that a numerical value such as the null accuracy was obtained. The LSTM-Bidirectional model exhibited the highest accuracy of classification and a recognition rate of 95.93%. The LSTM model exhibited the highest accuracy of detection at 98.09%. The classification accuracy of the LSTM model was 87.44%, which is relatively low. However, the detection rate of the LSTM-Bidirectional model was 97.9%, which was 0.19% less than that of the LSTM, and showed a high accuracy. The LSTM-Bidirectional model is excellent for both detection and recognition and is suitable for practical use. The next best performing model is the GRU-Bidirectional model. Respectively, the detection rate and recognition rate are 97.6% and 95.51%. There was a slight difference in the numerical accuracy, and, since the GRU-Bidirectional model exhibited the highest inference speed, it was judged that there was no problem in adopting the GRU-Bidirectional model for an application that requires a higher speed.

Table 5 presents a performance comparison based on the type and depth of each layer of the RNN. The distance between the sensor and the object is 20 cm. The detection performance of confirming the presence or absence of a metal object and the performance of classifying and recognizing five types of metal models were compared and analyzed.

Table 6 presents a performance comparison table for each layer type and depth of the measured RNN and a sensing speed (1 m/s, 3 m/s, 5 m/s); the distance between the sensor and the object is 20 cm. The detection performance of confirming the presence or absence of a metal object and the performance of classifying and recognizing five types of metal models were compared and analyzed.

Table 7 presents a performance comparison based on the type and depth of each layer of the RNN; the distance between the sensor and the object is 30 cm. The detection performance of confirming the presence or absence of a metal object and the performance of classifying and recognizing five types of metal models were compared and analyzed.

Table 8 presents a performance comparison table for each layer type and depth of the measured RNN and a sensing speed (1 m/s, 3 m/s, 5 m/s); the distance between the sensor and the object is 30 cm. The detection performance of confirming the presence or absence of a metal object and the performance of classifying and recognizing five types of metal models were compared and analyzed.

Table 9 presents a performance comparison table when the type and depth of each layer of the measured RNN; the distance between the sensor and the object is 40 cm. The detection performance of confirming the presence or absence of a metal object and the performance of classifying and recognizing five types of metal models were compared and analyzed.

Table 10 presents a performance comparison for each layer type and depth of the measured RNN and a sensing speed (1 m/s, 3 m/s, 5 m/s); the distance between the sensor and the object is 40 cm. The detection performance of confirming the presence or absence of a metal object and the performance of classifying and recognizing five types of metal models were compared and analyzed.

At a distance of 20 cm, the nine layers of the LSTM model exhibited the best detection performance, and the classification and recognition performance was the highest performance in the nine layers of the LSTM-Bidirectional model. The detection performance at a distance of 30 cm was the highest in the nine layers of the LSTM model similar to that at a distance of 20 cm, and the classification and recognition performance showed the highest performance in the nine layers of the LSTM-Bidirectional model. In addition, the detection performance at a distance of 40 cm was the highest in the nine layers of the LSTM model, and the classification and recognition performance showed the highest performance in the nine layers of the LSTM-Bidirectional model. As a result of learning the deep learning model, in general, the shallower the model layer, the lower the performance compared to other layers because of the irregularity in the sequence data and its incapability to learn the correlation between the front and rear signals. Conversely, the correlation between the input data in the feature extraction process is decreased due to the deeper layer of the model and the greater distance between its input and output ends. Our verification tests confirmed that all models are suitable for real-time detection and classification.

The overall performance was observed to be excellent for a distance and speed of 40 cm and 5 m/s, respectively.

The detection performance was better in the forward LSTM and GRU with general learning. It was confirmed that forward learning was more advantageous because detection judged similar patterns as a single signal rather than recognizing each similar pattern. In recognition and classification performance, LSTM- and GRU-Bidirectional learning with the reverse order of sequence data showed better performance compared to the forward LSTM and GRU that were trained normally, and the number of parameters was not increased. In addition, the detection performance of the interactive model was also high. Accordingly, it was confirmed that the interactive model showed better performance. Thus, it was deemed suitable for real-time data processing.

**Figure 16:**
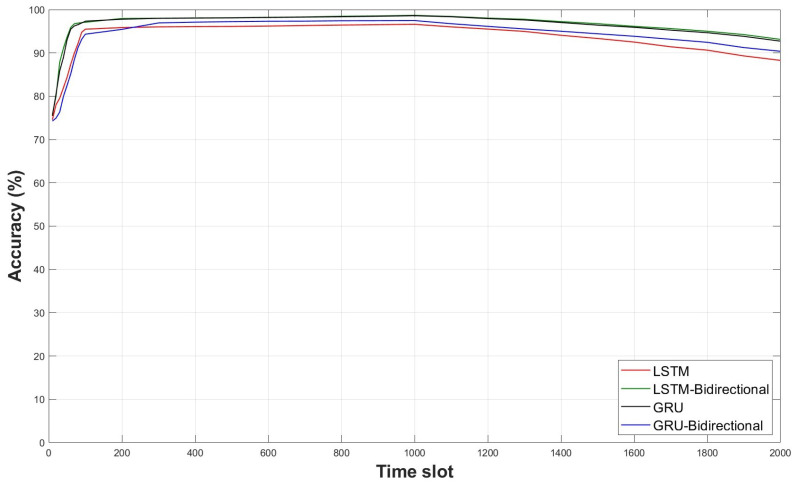
Comparison of each model accuracy by time slot length.

Figure 16 depicts the accuracy comparison according to data time series units for deep learning model training. The time series unit was varied from 10 to 2000 units. The signal acquisition frequency of the sensor used in this paper was about 200 Hz, and the time series unit for optimal learning was analyzed accordingly. The shorter the time slot, the more similar were the training results to the null accuracy of the dataset. Accuracy starts to converge from time slot 60 or higher, and it was confirmed that convergence was achieved at time slot 300. If the sampling rate of the sensor was exceeded and the time slot was increased, the ratio of null data to the training data was found to increase. The null dataset denotes a signal in a static state, and most of the signals were determined to be in the static state. As the time slot was increased, the proportion of the dynamic signal reduced, resulting in lower accuracy. In addition, as the time slot was increased, the inference cost of the deep learning model was also increased, and the inference time was increased. Therefore, it was advantageous to set the time slot to 60~300 for real-time inference of the deep learning model.

Table 11 compares the accuracy of the single prediction method and the overlap prediction method of the deep learning model. In the single prediction method, a large number of errors occurred due to the prediction of the next piece of data by skipping over the previously predicted data without processing them again. The overlap prediction method used in this paper predicts some of the previously predicted data from the current and next pieces of data and readjusts the prediction results through probability distribution. In this way, the error was minimized, and high accuracy was shown according to the overlapping sequence prediction results.

## 5. Discussion

In this paper, a deep learning model for detecting metal objects using MI sensors was compared, analyzed, and optimized. An RNN-based deep learning network was adopted, and the data acquired by the MI sensor was used as an input. RNN is a method mainly used for processing EMG and acoustic signals. Since the data of the MI sensor is also sequence data, it is possible to detect metal objects through learning. Unlike the EMG signal using a contact sensor, the MI sensor acquires data in a non-contact form. Therefore, the large amount of noise renders manual analysis difficult. Most of the existing signal detection algorithms detect the noise width of a signal and set a threshold value based on the peak value of the signal to be detected to perform detection. This method took a long time to set the threshold, and resetting this threshold was inconvenient when the environment changed. Deep learning minimizes this process, and the model learns to find rules independently from its acquired signals. In a fully refined situation, passive-based detection methods may be advantageous. However, in various environments and abnormal signals, a detection method based on deep learning is advantageous.

From our results, the LSTM-Unidirectional model demonstrated superior performance in detection tasks, while the LSTM-Bidirectional model excelled in classification tasks. When speed is a priority, substituting with the GRU model is beneficial. However, when accuracy is paramount, the LSTM model delivers higher performance.

A detection and classification method using deep learning-based MI sensor values has not yet been developed, and a model optimization process is required for development in this field. In this paper, we analyzed the number of layers and the number of layers that are advantageous for model optimization, as well as MI sensors under various conditions. In addition, we succeeded in increasing the accuracy by processing the sequence length of the input data and performing additional work in the prediction process.

## 6. Conclusions

In this study, a deep learning model was devised to detect metal objects using a 4 × 4 precision arrangement of AICHI AMI306 sensors. This model’s performance was rigorously compared and analyzed. We investigated the efficacy of both the LSTM and the GRU layers of the RNN model, considering both forward learning and bidirectional learning. The optimal number of layers was discerned by contrasting performances at varying layer depths. Moreover, the optimal length for training the deep learning network was determined by altering the length of the input data. While our model showcases promising results, it is imperative to acknowledge that the overlap prediction method’s performance was solely verified by juxtaposing it against the accuracy of the method used in this study. Such a comparison might not encapsulate the entirety of the potential methods available. Anticipated trajectories for further research include the development of a novel model to analyze the MI sensor’s values, leveraging the groundwork of optimization delineated in this paper. There is a pressing need to venture into data augmentation techniques, especially harnessing the capabilities of generative adversarial networks (GANs), to supplement datasets that may be sparse. The findings from this research set a precedent for MI sensor-based detection using deep learning. As the realm of MI sensors broadens in application, the optimized techniques presented here can pave the way for more accurate and efficient implementations in real-world scenarios.

## Figures and Tables

**Table 1:** Size and weight of the metal plate.

Type	Weight (kg)	Size
Plate A	0.039	50 mm × 50 mm, 2 T
Plate B	0.157	100 mm × 100 mm, 2 T
Plate C	1.413	300 mm × 300 mm, 2 T
Plate D	3.925	500 mm × 500 mm, 2 T
Plate E	7.693	700 mm × 700 mm, 2 T

**Table 2:** Experimental environment specifications.

Type	Environment
OS	Ubuntu 20.04
Language	Python 3.8
Library	Tensorflow 2.4, Numpy
CPU	AMD Ryzen 7 3700X 8-Core Processor
Memory	64 GB

**Table 3:** Comparison of parameter counts and inference speed across models based on layer quantities.

	LSTM		LSTM-Bidirectional		GRU		GRU-Bidirectional	
	Parameter	Speed	Parameter	Speed	Parameter	Speed	Parameter	Speed
**1 layer**	16,908	0.72 ms	8716	0.70 ms	12,876	0.70 ms	6732	0.69 ms
**3 layer**	83,334	0.96 ms	58,758	0.86 ms	63,174	0.84 ms	44,742	0.80 ms
**5 layer**	149,382	1.11 ms	108,422	0.98 ms	113,094	0.99 ms	82,374	0.92 ms
**7 layer**	215,430	1.27 ms	158,086	1.15 ms	163,014	1.12 ms	120,006	1.05 ms
**9 layer**	281,478	1.47 ms	207,750	1.25 ms	212,934	1.24 ms	157,638	1.11 ms

**Table 4:** Comparison of overall inference accuracy for each model.

	LSTM		LSTM-Bidirectional		GRU		GRU-Bidirectional	
	Detection	Classification	Detection	Classification	Detection	Classification	Detection	Classification
**1 layer**	86.05%	66.69%	81.77%	66.63%	86.28%	66.69%	81.80%	66.69%
**3 layer**	96.95%	80.04%	96.37%	83.81%	95.99%	77.01%	94.55%	79.88%
**5 layer**	97.32%	83.67%	96.85%	91.01%	97.07%	83.02%	95.88%	89.44%
**7 layer**	97.49%	85.72%	97.66%	94.80%	97.27%	85.23%	96.25%	92.59%
**9 layer**	**98.09%**	87.44%	97.90%	**95.93%**	97.44%	85.22%	97.60%	95.51%

**Table 5:** Comparison of inference accuracy across different models and layers at a 20 cm distance.

20 cm	LSTM		LSTM-Bidirectional		GRU		GRU-Bidirectional	
	Detection	Classification	Detection	Classification	Detection	Classification	Detection	Classification
**1 layer**	85.79%	68.09%	81.30%	68.02%	85.16%	68.09%	80.16%	68.09%
**3 layer**	97.18%	83.68%	96.44%	89.70%	96.00%	78.43%	94.70%	87.11%
**5 layer**	97.44%	85.15%	97.03%	93.60%	96.98%	84.63%	95.58%	92.35%
**7 layer**	97.51%	87.14%	97.88%	95.27%	97.02%	85.91%	95.91%	90.52%
**9 layer**	**98.30%**	87.91%	97.90%	**96.03%**	97.70%	87.63%	97.77%	95.32%

**Table 6:** Analysis of inference accuracy across models, layers, and speeds at a 20 cm distance.

**1 m/s**	**LSTM**		**LSTM-Bidirectional**		**GRU**		**GRU-Bidirectional**	
	**Detection**	**Classification**	**Detection**	**Classification**	**Detection**	**Classification**	**Detection**	**Classification**
**1 layer**	82.03%	72.96%	77.78%	72.75%	79.28%	72.96%	75.50%	72.96%
**3 layer**	96.34%	80.18%	96.10%	85.95%	94.71%	78.45%	92.85%	82.98%
**5 layer**	97.65%	83.05%	97.09%	90.66%	96.30%	82.28%	94.70%	87.63%
**7 layer**	97.41%	84.20%	97.96%	93.02%	96.66%	83.57%	94.72%	88.23%
**9 layer**	**98.33%**	85.43%	97.78%	**94.43%**	97.70%	84.85%	97.90%	92.40%
**3 m/s**	**LSTM**		**LSTM-Bidirectional**		**GRU**		**GRU-Bidirectional**	
	**Detection**	**Classification**	**Detection**	**Classification**	**Detection**	**Classification**	**Detection**	**Classification**
**1 layer**	87.13%	64.01%	81.05%	64.01%	87.27%	64.01%	79.12%	64.01%
**3 layer**	97.62%	83.07%	96.08%	91.10%	96.25%	77.05%	93.49%	88.07%
**5 layer**	96.89%	83.65%	96.58%	93.28%	97.24%	82.64%	95.63%	92.90%
**7 layer**	97.74%	87.31%	97.87%	96.18%	97.20%	84.35%	96.40%	90.77%
**9 layer**	**98.15%**	86.30%	97.63%	**96.53%**	97.74%	86.86%	97.85%	96.10%
**5 m/s**	**LSTM**		**LSTM-Bidirectional**		**GRU**		**GRU-Bidirectional**	
	**Detection**	**Classification**	**Detection**	**Classification**	**Detection**	**Classification**	**Detection**	**Classification**
**1 layer**	88.22%	67.30%	85.07%	67.30%	88.93%	67.30%	85.86%	67.30%
**3 layer**	97.58%	87.79%	97.14%	92.05%	96.94%	79.79%	95.88%	90.27%
**5 layer**	97.76%	88.74%	97.42%	96.85%	97.39%	88.97%	96.42%	96.53%
**7 layer**	97.38%	90.72%	97.80%	96.61%	97.20%	89.80%	96.60%	92.56%
**9 layer**	**98.41%**	91.99%	98.30%	97.13%	97.64%	91.20%	97.56%	**97.36%**

**Table 7:** Comparison of inference accuracy across different models and layers at a 30 cm distance.

30 cm	LSTM		LSTM-Bidirectional		GRU		GRU-Bidirectional	
	Detection	Classification	Detection	Classification	Detection	Classification	Detection	Classification
**1 layer**	85.72%	66.23%	81.72%	66.21%	85.99%	66.23%	81.91%	66.23%
**3 layer**	96.83%	81.17%	96.31%	84.22%	96.21%	78.27%	94.61%	78.83%
**5 layer**	97.35%	83.98%	96.67%	93.50%	97.03%	83.34%	95.77%	90.62%
**7 layer**	97.43%	85.99%	97.47%	95.38%	97.21%	85.79%	96.29%	94.04%
**9 layer**	**97.95%**	87.51%	97.80%	**96.31%**	97.41%	84.95%	97.59%	96.19%

**Table 8:** Analysis of inference accuracy across models, layers, and speeds at a 30 cm distance.

**1 m/s**	**LSTM**		**LSTM-Bidirectional**		**GRU**		**GRU-Bidirectional**	
	**Detection**	**Classification**	**Detection**	**Classification**	**Detection**	**Classification**	**Detection**	**Classification**
**1 layer**	80.47%	71.06%	78.42%	71.00%	80.45%	71.06%	75.38%	71.06%
**3 layer**	95.28%	77.68%	94.61%	81.86%	93.96%	77.60%	92.37%	78.67%
**5 layer**	96.53%	83.57%	95.83%	89.78%	95.76%	80.98%	94.15%	85.50%
**7 layer**	95.99%	85.02%	96.84%	92.05%	95.91%	82.92%	94.85%	91.04%
**9 layer**	97.15%	85.89%	**97.52%**	94.26%	96.31%	81.65%	97.43%	**94.85%**
**3 m/s**	**LSTM**		**LSTM-Bidirectional**		**GRU**		**GRU-Bidirectional**	
	**Detection**	**Classification**	**Detection**	**Classification**	**Detection**	**Classification**	**Detection**	**Classification**
**1 layer**	87.96%	62.29%	81.13%	62.29%	87.20%	62.29%	81.00%	62.29%
**3 layer**	97.34%	78.43%	96.91%	85.83%	97.06%	75.98%	95.09%	76.54%
**5 layer**	97.38%	82.83%	97.00%	94.46%	97.56%	81.45%	96.62%	90.87%
**7 layer**	97.90%	86.80%	97.59%	96.49%	97.45%	83.65%	96.95%	94.34%
**9 layer**	**98.27%**	86.11%	97.80%	**96.60%**	97.91%	83.27%	97.75%	96.03%
**5 m/s**	**LSTM**		**LSTM-Bidirectional**		**GRU**		**GRU-Bidirectional**	
	**Detection**	**Classification**	**Detection**	**Classification**	**Detection**	**Classification**	**Detection**	**Classification**
**1 layer**	88.73%	65.35%	85.62%	65.35%	89.98%	65.35%	89.35%	65.35%
**3 layer**	97.85%	87.40%	97.39%	84.96%	97.61%	81.25%	96.38%	81.29%
**5 layer**	98.13%	85.53%	97.19%	96.24%	97.77%	87.59%	96.55%	95.48%
**7 layer**	98.39%	86.15%	97.98%	97.59%	98.27%	90.80%	97.08%	96.76%
**9 layer**	**98.43%**	90.53%	98.07%	**98.09%**	98.02%	89.93%	97.60%	97.71%

**Table 9:** Comparison of inference accuracy across different models and layers at a 40 cm distance.

40 cm	LSTM		LSTM-Bidirectional		GRU		GRU-Bidirectional	
	Detection	Classification	Detection	Classification	Detection	Classification	Detection	Classification
**1 layer**	86.67%	66.03%	82.19%	65.93%	87.57%	66.03%	83.01%	66.03%
**3 layer**	96.89%	76.01%	96.38%	78.70%	95.75%	74.62%	94.86%	75.15%
**5 layer**	97.19%	82.18%	96.89%	86.46%	97.19%	81.40%	96.22%	85.94%
**7 layer**	97.54%	84.09%	97.69%	9 3.84%	97.52%	84.14%	96.48%	92.79%
**9 layer**	**98.05%**	86.99%	98.01%	**95.46%**	97.26%	83.56%	97.47%	94.98%

**Table 10:** Analysis of inference accuracy across models, layers, and speeds at a 40 cm distance.

**1 m/s**	**LSTM**		**LSTM-Bidirectional**		**GRU**		**GRU-Bidirectional**	
	**Detection**	**Classification**	**Detection**	**Classification**	**Detection**	**Classification**	**Detection**	**Classification**
**1 layer**	82.03%	71.18%	78.97%	70.86%	81.55%	71.18%	76.74%	71.18%
**3 layer**	95.27%	75.30%	95.47%	78.83%	94.80%	75.72%	92.92%	76.94%
**5 layer**	96.74%	81.88%	96.49%	85.46%	96.28%	80.70%	95.38%	80.61%
**7 layer**	97.39%	86.77%	97.66%	91.05%	96.78%	82.18%	95.39%	87.92%
**9 layer**	97.65%	86.07%	**97.88%**	92.58%	96.20%	80.58%	97.49%	**93.06%**
**3 m/s**	**LSTM**		**LSTM-Bidirectional**		**GRU**		**GRU-Bidirectional**	
	**Detection**	**Classification**	**Detection**	**Classification**	**Detection**	**Classification**	**Detection**	**Classification**
**1 layer**	88.43%	61.49%	80.97%	61.49%	89.03%	61.49%	81.07%	61.49%
**3 layer**	97.65%	74.06%	96.85%	78.35%	96.42%	71.98%	95.23%	72.68%
**5 layer**	96.94%	79.42%	96.90%	85.89%	97.44%	79.29%	96.79%	88.19%
**7 layer**	97.43%	83.47%	97.46%	93.80%	97.69%	81.79%	97.21%	94.62%
**9 layer**	**98.47%**	84.61%	97.94%	**96.66%**	97.63%	81.37%	97.78%	95.57%
**5 m/s**	**LSTM**		**LSTM-Bidirectional**		**GRU**		**GRU-Bidirectional**	
	**Detection**	**Classification**	**Detection**	**Classification**	**Detection**	**Classification**	**Detection**	**Classification**
**1 layer**	89.55%	65.43%	86.63%	65.43%	92.13%	65.43%	91.21%	65.43%
**3 layer**	97.75%	78.67%	96.82%	78.93%	96.04%	76.16%	96.44%	75.84%
**5 layer**	97.89%	85.26%	97.29%	88.02%	97.84%	84.22%	96.50%	89.02%
**7 layer**	97.81%	82.02%	97.94%	96.67%	98.08%	88.46%	96.84%	95.83%
**9 layer**	98.04%	90.28%	**98.20%**	**97.13%**	97.96%	88.73%	97.15%	96.30%

**Table 11:** Comparison of inference accuracy for each model and layer (distance = 40 cm).

	LSTM		LSTM-Bidirectional		GRU		GRU-Bidirectional	
	Detection	Classification	Detection	Classification	Detection	Classification	Detection	Classification
**Single (9 layer)**	**98.09%**	87.44%	97.90%	**95.93%**	97.44%	85.22%	97.60%	95.51%
**Nested (9 layer)**	**99.23%**	90.73%	99.12%	**98.07%**	98.97%	89.41%	99.14%	97.88%

## Data Availability

Data are contained within the article.

## References

[1] Goyal S., Benjamin P. (2014). Object Recognition Using Deep Neural Networks: A Survey. arXiv.

[2] Bansal M., Kumar M., Kumar M. (2020). 2D Object Recognition Techniques: State-of-the-Art Work. Arch. Comput. Methods Eng..

[3] Liu X., Song L., Liu S., Zhang Y. (2021). A Review of Deep-Learning-Based Medical Image Segmentation Methods. Sustainability.

[4] Wang W., Zhou T., Porikli F., Crandall D., Van Gool L. (2021). A Survey on Deep Learning Technique for Video Segmentation. arXiv.

[5] Zhang Y., Sidibé D., Morel O., Mériaudeau F. (2021). Deep multimodal fusion for semantic image segmentation: A survey. Image Vis. Comput..

[6] Zaidi S.S.A., Ansari M.S., Aslam A., Kanwal N., Asghar M., Lee B. (2022). A Survey of Modern Deep Learning based Object Detection Models. Digit. Signal Process..

[7] Liu Y., Sun P., Wergeles N., Shang Y. (2021). A survey and performance evaluation of deep learning methods for small object detection. Expert Syst. Appl..

[8] Zou Z., Shi Z., Guo Y., Ye J. (2019). Object Detection in 20 Years: A Survey. arXiv.

[9] Liu L., Ouyang W., Wang X., Fieguth P., Chen J., Liu X., Pietikäinen M. (2020). Deep Learning for Generic Object Detection: A Survey. Int. J. Comput. Vis..

[10] Borji A., Cheng M.-M., Hou Q., Jiang H., Li J. (2014). Salient Object Detection: A Survey. arXiv.

[11] Kim H., Kwon S., Lee S. (2021). NRA-Net—Neg-Region Attention Network for Salient Object Detection with Gaze Tracking. Sensors.

[12] Dang Q., Yin J., Wang B., Zheng W. (2019). Deep learning based 2D human pose estimation: A survey. Tsinghua Sci. Technol..

[13] Marchand E., Uchiyama H., Spindler F. (2016). Pose Estimation for Augmented Reality: A Hands-On Survey. IEEE Trans. Vis. Comput. Graph..

[14] Chen Y., Tian Y., He M. (2020). Monocular Human Pose Estimation: A Survey of Deep Learning-based Methods. arXiv.

[15] Erol A., Bebis G., Nicolescu M., Boyle R.D., Twombly X. (2007). Vision-based hand pose estimation: A review. Comput. Vis. Image Underst..

[16] Doosti B. (2019). Hand Pose Estimation: A Survey. arXiv.

[17] Hjelmås E., Low B.K. (2001). Face Detection: A Survey. Comput. Vis. Image Underst..

[18] Kumar A., Kaur A., Kumar M. (2018). Face detection techniques: A review. Artif. Intell. Rev..

[19] Yang S., Luo P., Loy C.C., Tang X. (2015). WIDER FACE: A Face Detection Benchmark. IEEE Trans. Pattern Anal. Mach. Intell..

[20] Rowley H.A., Baluja S., Kanade T. (1998). Neural network-based face detection. IEEE Trans. Pattern Anal. Mach. Intell..

[21] Hsu R., Abdel-Mottaleb M., Jain A.K. (2002). Face detection in color images. IEEE Trans. Pattern Anal. Mach. Intell..

[22] Candamo J., Shreve M., Goldgof D.B., Sapper D.B., Kasturi R. (2010). Understanding Transit Scenes: A Survey on Human Behavior-Recognition Algorithms. IEEE Trans. Intell. Transp. Syst..

[23] Popoola O.P., Wang K. (2012). Video-Based Abnormal Human Behavior Recognition–A Review. IEEE Trans. Syst. Man Cybern. Part C.

[24] Chandolikar N., Joshi C., Roy P., Gawas A., Vishwakarma M. Voice Recognition: A Comprehensive Survey. Proceedings of the 2022 International Mobile and Embedded Technology Conference (MECON).

[25] Liu W., Liu J., Hao C., Gao Y., Wang Y.-L. (2022). Multichannel adaptive signal detection: Basic theory and literature review. Sci. China Inf. Sci..

[26] Kassam S.A., Poor H.V. (1985). Robust techniques for signal processing: A survey. Proc. IEEE.

[27] Lakshmi M.R., Prasad T.V., Prakash D.V.C. (2014). Survey on EEG signal processing methods. Int. J. Adv. Res. Comput. Sci. Softw. Eng..

[28] Xie D., Zhang L., Bai L. (2017). Deep Learning in Visual Computing and Signal processing. Appl. Comput. Intell. Soft Comput..

[29] Geng Z., Yan H., Zhang J., Zhu D. (2021). Deep-Learning for Radar: A Survey. IEEE Access.

[30] Ha S., Lee D., Kim H., Kwon S., Kim E., Yang J., Lee S. (2021). Neural Network for Metal Detection Based on Magnetic Impedance Sensor. Sensors.

[31] Wang T., Zhou Y., Lei C., Luo J., Xie S., Pu H. (2017). Magnetic impedance biosensor: A review. Biosens. Bioelectron..

[32] MI Sensor | Smart Company | Aichi Steel Corporation. https://www.aichi-steel.co.jp/ENGLISH/smart/mi/.

[33] Mohri K., Uchiyama T., Shen L.P., Cai C.M., Panina L.V. (2001). Sensitive micro magnetic sensor family utilizing magneto-impedance (MI) and stress-impedance (SI) effects for intelligent measurements and controls. Sensors Actuators A Phys..

[34] Hika K., Panina L.V., Mohri K. (1996). Magneto-impedance in sandwich film for magnetic sensor heads. IEEE Trans. Magn..

[35] Zaremba W., Sutskever I., Vinyals O. (2014). Recurrent Neural Network Regularization. arXiv.

[36] Mikolov T., Karafiát M., Burget L., Cernocký J., Khudanpur S. (2010). Recurrent neural network based language model. Interspeech.

[37] Medsker L., Jain L.C. (1999). Recurrent Neural Networks: Design and Applications.

[38] Mikolov T., Kombrink S., Burget L., Cernocky J., Khudanpur S. Extensions of recurrent neural network language model. Proceedings of the 2011 IEEE International Conference on Acoustics, Speech and Signal Processing (ICASSP).

[39] Gregor K., Danihelka I., Graves A., Rezende D., Wierstra D. Draw: A recurrent neural network for image generation. Proceedings of the International Conference on Machine Learning.

[40] Donkers T., Loepp B., Ziegler J. Sequential User-based Recurrent Neural Network Recommendations. Proceedings of the Eleventh ACM Conference on Recommender Systems.

[41] Mandic D., Chambers J. (2001). Recurrent Neural Networks for Prediction: Learning Algorithms, Architectures and Stability.

[42] Giles C.L., Lawrence S., Tsoi A.C. (2001). Noisy Time Series Prediction using Recurrent Neural Networks and Grammatical Inference. Mach. Learn..

[43] Hochreiter S., Schmidhuber J. (1997). Long Short-Term Memory. Neural Comput..

[44] Van Houdt G., Mosquera C., Nápoles G. (2020). A review on the long short-term memory model. Artif. Intell. Rev..

[45] Graves A. (2012). Long Short-Term Memory. Stud. Comput. Intell..

[46] Chung J., Gulcehre C., Cho K., Bengio Y. (2014). Empirical Evaluation of Gated Recurrent Neural Networks on Sequence Modeling. arXiv.

[47] Dey R., Salem F.M. Gate-variants of Gated Recurrent Unit (GRU) neural networks. Proceedings of the 2017 IEEE 60th International Midwest Symposium on Circuits and Systems (MWSCAS).

[48] Zhao R., Wang D., Yan R., Mao K., Shen F., Wang J. (2018). Machine Health Monitoring Using Local Feature-Based Gated Recurrent Unit Networks. IEEE Trans. Ind. Electron..

[49] Collis R.T.H. (1970). Lidar. Appl. Opt..

[50] Dong P., Chen Q. (2017). LiDAR Remote Sensing and Applications.

